# Combined transcatheter managements of a huge spontaneous iliac pseudoaneurysm presenting with fever of unknown origin: a case report

**DOI:** 10.1186/1752-1947-8-118

**Published:** 2014-04-06

**Authors:** Shuang Li, Dao-Jing Huang, Kai Gong, Ya-Wei Xu

**Affiliations:** 1 Department of Cardiology, Shanghai Tenth People’s Hospital, Tongji University School of Medicine, No. 301 Yanchang Middle Road, Shanghai 200072, China; 2 Group of Vascular Surgery, Department of General Surgery, Shanghai Tongji Hospital, Tongji University School of Medicine, Shanghai 200065, China

## Abstract

**Introduction:**

We present a successful combined endovascular repair of a rare huge spontaneous pseudoaneurysm in a patient troubled solely with fever of unknown origin.

**Case presentation:**

A 79-year-old Chinese man complained of repeated episodes of fever for 10 days. His medical history, physical examination and laboratory tests were not significant. Routine antibiotics were given for suspected sepsis lasting 4 weeks without clinical improvement. Finally, an 81.9×61.6mm iliac pseudoaneurysm was found. The pseudoaneurysm originated from his left iliac arteries and covered the bifurcation of the left common iliac artery and proximal ends of both internal and external iliac arteries. A combination of endovascular repair with coil embolization and stent graft implantation was successfully performed. He underwent an uneventful recovery.

**Conclusions:**

Spontaneous pseudoaneurysm with fever of unknown origin should not be ignored, especially for patients with a high risk for atherosclerosis. Combined transcatheter managements might be an alternative approach to deal with complex pseudoaneurysms, effectively and safely.

## Introduction

Pseudoaneurysm (PA) or false aneurysm is a relatively rare but threatening clinical disease [1]. PAs are always the results of trauma [2], inflammation [3] or iatrogenic procedures [4,5], presenting with pulsatile masses, compressive feelings or hemorrhage as the most notable manifestations [1]. We report a case of a patient troubled with fever of unknown origin (FUO) that was solely due to the spontaneous formation of a huge PA in the bifurcation of his left common iliac artery. Combined endovascular managements using coils, balloon and stent graft were successfully performed.

## Case presentation

**Figure 1:**
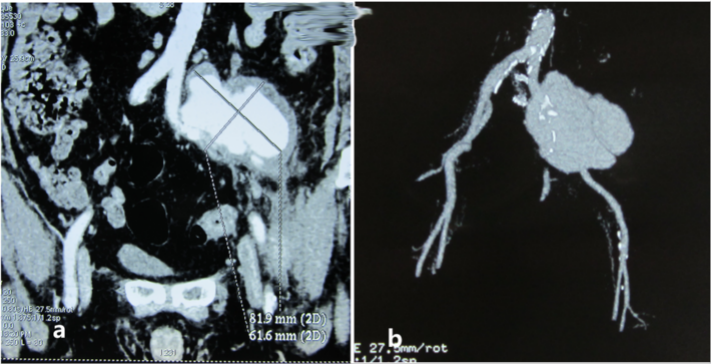
**Imaging of computed tomographic angiography before the endovascular procedure. (a)** and **(b)** The contrast-enhanced computed tomographic angiography showed an 81.9×61.6mm lobular hematoma covering the bifurcation of the left common iliac artery and the proximal ends of both internal and external iliac arteries.

A 79-year-old Chinese man was admitted to our hospital complaining of episodes of repeated fever and some uncertain discomfort for 10 days. He had a nonproductive cough, but no chill, pharyngalgia, arthralgia, chest pain, dyspnea or diarrhea. He denied experience of trauma or drug abuse. He previously had been well but had a medical history of hypercholesterolemia and a long duration of two packs/day cigarette smoking. His father died from a sudden heart attack and he also had two brothers suffering from hypertension. On physical examination, he was 69 inches (1.73m) tall and weighed 178lb (81kg). He was febrile with a heart rate of 90 beats per minute, blood pressure 135/85mmHg, and respiratory rate 22 breaths per minute. Head and neck examinations were significant for pale conjunctivae and a left carotid bruit. His chest was clear to auscultation. His heart rhythm was regular with a nondisplaced apical impulse, an S4 gallop, no murmurs or rubs. His abdomen was benign, with no tenderness or masses. He had symmetrical palpable pulses in the femoral, popliteal and pedal arteries bilaterally without changes of skin appearance, temperature or sensations. Laboratory assessments revealed a high level of white blood cell count (20.7×10^9^/L), neutrophils (85%), C-reactive protein (132mg/L), erythrocyte sedimentation rate (45mm/hour) and a low level of hemoglobin (94g/L). A lipid level test showed total cholesterol, triglycerides, high-density lipoprotein and low-density lipoprotein were 342, 280, 35 and 263mg/dL respectively. Liver and kidney functions, urine and stool tests were normal. A chest X-ray showed no recent infections. Conventional antibiotics using cefoxitin and levofloxacin were given for 10 days in the out-patient department and 4 days in our hospital for suspected sepsis, but his temperature still fluctuated and mostly remained above 38°C. However, bacteriological cultures of blood, urine, and sputum were negative. Immune markers like complements, interleukins, self-antibodies and globulins showed no abnormalities. Tumor and human immunodeficiency virus biomarkers were also negative. However, a brief scanning of computed tomography (CT) and magnetic resonance imaging (MRI) were done to search for the focus of the fever and suggested edemas and abnormalities around his left greater psoas and peritoneum, suggesting inflammatory changes. Ceftizoxime and clindamycin were regulated to use on advice from microbiologists and kept for another 2 weeks, but still without any conspicuous relief of symptoms. Based on the ambiguity of the CT and MRI done earlier, a contrast-enhanced computed tomographic angiography ([CTA], Figures 1a and 1b) was performed and demonstrated the existence of an 81.9mm×61.6mm PA, which originated from his left iliac arteries and covered the bifurcation of his left common iliac artery and proximal ends of both his internal and external iliac arteries.

**Figure 2:**
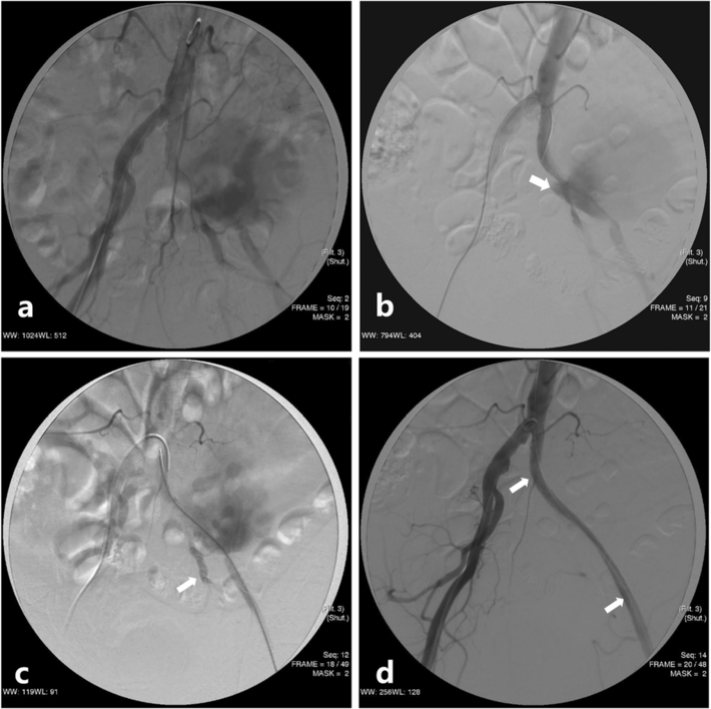
**Imaging of angiographies during the endovascular procedure. (a)** Preprocedural selective left iliac angiogram confirmed the existence of a huge pseudoaneurysm from the left iliac artery and **(b)** clearly revealed the orifice (arrow) of the pseudoaneurysm with a wide neck located at the bifurcation of the left common iliac artery. **(c)** Metal coils (arrow) were fixed at the distal end of the left internal iliac artery. **(d)**. Postprocedural selective angiography demonstrated complete exclusion of pseudoaneurysm with a self-expandable covered stent (arrows marked the proximal and the distal ends respectively).

The endovascular procedure was performed 1 day after the CTA. Heparin 4000IU was administered intra-arterially once access had been obtained. A 6-F artery sheath punctured his contralateral femoral artery. Then a 5-F Rösch inferior mesenteric catheter (Cordis) and 0.035 3mm “J” standard guide wire (Cook) were used together to enter his left common iliac artery. Hand injections of contrast demonstrated the existence of a huge PA (Figure 2a) and also identified both the rupture of PA and the proximal end of his left internal iliac artery (Figure 2b). Another catheter and guide wire were inserted through an ipsilateral femoral artery puncture. Via the catheter passway, metal coils (Cook: 8mm diameter, Figure 2c) were released and fixed at the distal end of his left internal iliac artery. Then the left femoral artery was exposed and a 14-F artery catheter (Cordis) was inserted. A 14mm×7cm Bard self-expandable polytetrafluoroethylene-covered Fluency stent (Boston Scientific) was placed after dilating with an 8mm×4cm angioplasty balloon. A selective left iliac arteriography (Figure 2d) 5 minutes after deployment of the stent graft confirmed complete exclusion of the PA and normal flow to the arteries of his ipsilateral lower extremity.

**Figure 3:**
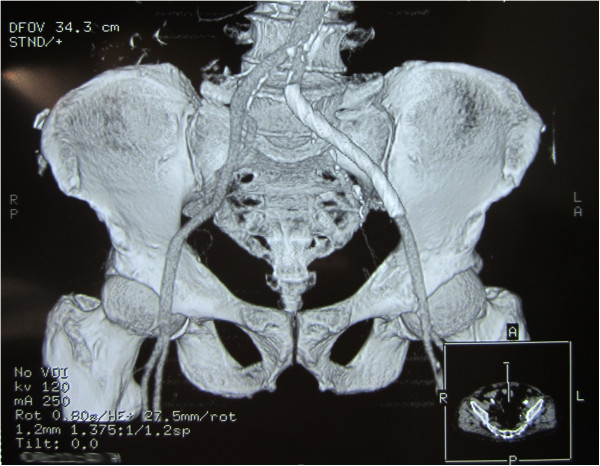
**Imaging of computed tomographic angiography after 30 days of discharge.** Contrast-enhanced computed tomographic angiography after 30 days of the treatment revealed a well-expanded stent and total occlusion of pseudoaneurysm.

The postprocedural course went well. The patient received subcutaneous low-molecular-weight heparin, urokinase and prophylactic cefminox. Furthermore, he was given long-term medications for hypercholesterolemia with atorvastatin, aspirin and omega 3 cod liver oil. Initially there was eradication of fever. His subsequent clinical course was uneventful and he was discharged 7 days after the endovascular treatment. CTA performed after 30 days showed a well-expanded stent with no signs of a new PA (Figure 3) and a decreasing and consolidated residual hematoma. He received oral administration of warfarin for 3-month anticoagulation, maintaining an international normalized ratio of 2.0 to 3.0. At 1.5-year clinical follow-up, his recovery was good.

## Conclusions

Isolated PAs are always secondary to blunt trauma [2], inflammation [3], and iatrogenic procedures such as organ transplantation [4], interventional procedures [5] or neoplasia [6] that cause laceration of part of the vessel wall and extravasation of blood into surrounding tissues, followed by tamponade and clot formation. Typically, PAs present with pulsatile masses, compressive symptoms, secondary hemorrhage and neurologic deficit as the most common clinical manifestations. However, even spontaneous PAs or PAs without clear origins are clinically rare; they have been reported to occur in the facial artery [7], tibioperoneal trunk and anterior tibial artery [6], lumbar artery [8] and superficial femoral artery [9], presenting as pulsatile masses in the former two cases and as ruptured hemorrhage in the latter two respectively.

FUO is defined as a temperature higher than 38.3°C on several occasions and lasting longer than 3 weeks, with a diagnosis that remains uncertain after 1 week of investigation. There are well over 200 different reported causes of FUO and five major categories of conditions: infections, neoplasms, connective tissue diseases, miscellaneous disorders and undiagnosed conditions [10,11].

To the best of our knowledge, spontaneous formations of PAs originating from iliac arteries with atypical clinical manifestations are extremely rare. The mechanism of this case may be correlated with iliac arteriosclerosis. Underlying brittleness of the arterial wall due to extensive lesions of calcification (Figures 1a and 1b) both in the abdominal aorta and bilateral iliac arteries made them predisposed to rupture. Some cases of PAs were reported to be associated with the process of calcification or atherosclerosis [12-17]. Other than images, the patient’s high body mass index, cigarette smoking, family history of heart attack and medical history of hypercholesterolemia all contribute to risk factors for arteriosclerosis. Besides, in this case the deep location and pelvic surroundings made the PA present without a remarkable pulsatile mass or ruptured hemorrhage.

As for treatments of PAs, in recent years, several interventional techniques have been developed to achieve occlusion of PAs, including ultrasound-guided compression, direct percutaneous thrombin injection, transcatheter embolization with coils, glue, gelfoam or sclerosing agents and stent graft placement, showing a shorter hospitalization time, more favorable success rates and minimal morbidity [1]. Although traditional opening surgery may be necessary in some instances, the pendulum has now swung to endovascular therapy [2].

In this case, the PA is a complex condition as some episodes of giant size, wide neck and bifurcation lesion existed simultaneously. Considering those situations, we tend to opt for combined endovascular managements sequentially. Embolization of his internal artery with coils ensured no recurrence of blood flow into the PA, and then occlusion of the ruptured PA and support of the vessel that supplied blood to his lower extremity with a self-expandable covered stent graft were performed successfully. The result of a 1.5-year follow-up confirmed the combined treatment to be effective and safe.

## Consent

Written informed consent was obtained from the patient for publication of this case report and any accompanying images. A copy of the written consent is available for review by the Editor-in-Chief of this journal.

## Abbreviations

CT: Computed tomography; CTA: Computed tomographic angiography; FUO: Fever of unknown origin; MRI: Magnetic resonance imaging; PA: Pseudoaneurysm.

## Competing interests

The authors declare that they have no competing interests.

## Authors’ contributions

SL participated in the design of the study and wrote the paper. DJH participated in the treatment and helped write the paper. KG participated in the treatment. YWX conceived of the study, and participated in its design and coordination and helped to draft the manuscript. All authors read and approved the final manuscript.
